# Atypical parathyroid adenoma: clinical and anatomical pathologic features

**DOI:** 10.1186/s12957-021-02123-7

**Published:** 2021-01-20

**Authors:** Alessandro Galani, Riccardo Morandi, Mira Dimko, Sarah Molfino, Carla Baronchelli, Silvia Lai, Federico Gheza, Carlo Cappelli, Claudio Casella

**Affiliations:** 1 Department of Clinical and Experimental Sciences, Surgical Clinic, University of Brescia, Brescia, Italy; 2 Nephrology and Dialysis Unit, ASST Carlo Poma, Mantova, Italy; 3 Institute of Pathology, Spedali Civili, Brescia, Italy; 4 Department of Translation and Precision Medicine, Sapienza University of Rome, Rome, Italy; 5 Department of Clinical and Experimental Sciences, Unit of Endocrinology and Metabolism, University of Brescia, Brescia, Italy; 6 Department of Molecular and Translational Medicine, Surgical Clinic, University of Brescia, Brescia, Italy

**Keywords:** Atypical parathyroid adenoma, Typical adenoma, Parathyroid carcinoma, Minimally invasive parathyroidectomy, Primary hyperparathyroidism

## Abstract

**Background:**

Primary hyperparathyroidism is an endocrine pathology that affects calcium metabolism. Patients with primary hyperparathyroidism have high concentrations of serum calcium or high concentrations of parathyroid hormone, or incorrect parathyroid hormone levels for serum calcium values. Primary hyperparathyroidism is due to the presence of an adenoma/single-gland disease in 80–85%. Multiple gland disease or hyperplasia accounts for 10–15% of cases of primary hyperparathyroidism. Atypical parathyroid adenoma and parathyroid carcinoma are both responsible for about 1.2–1.3% and 1% or less of primary hyperparathyroidism, respectively.

**Methods:**

We performed a retrospective cohort study and enrolled 117 patients with primary hyperparathyroidism undergoing minimally invasive parathyroidectomy. Histological and immunohistochemical examination showed that 107 patients (91.5%) were diagnosed with typical adenoma (group A), while 10 patients (8.5%) were diagnosed with atypical parathyroid adenoma (group B). None of the patients were affected by parathyroid carcinoma.

**Results:**

Significant statistical differences were found in histological and immunohistochemical parameters as pseudocapsular invasion (*p* <  0.001), bands of fibrosis (*p* <  0.001), pronounced trabecular growth (*p* <  0.001), mitotic rates of > 1/10 high-power fields (HPFs) (*p* <  0.001), nuclear pleomorphism (*p* = 0.036), thick capsule (*p* <  0.001), Ki-67+ > 4% (*p* <  0.001), galectin-3 + (*p* = 0.002), and protein gene product (PGP) 9.5 + (*p* = 0.038).

**Conclusions:**

Atypical parathyroid adenoma is a tumor that has characteristics both of typical adenoma and parathyroid carcinoma. The diagnosis is reached by excluding with strict methods the presence of malignancy criteria. Atypical parathyroid adenoma compared to typical adenoma showed significant clinical, hematochemical, histological, and immunohistochemical differences. We did not find any disease relapse in the 10 patients with atypical parathyroid adenoma during 60 months of follow-up time.

## Introduction

Primary hyperparathyroidism (PHPT) is an endocrine pathology that affects calcium metabolism. Patients with PHPT have high concentrations of serum calcium or high concentrations of parathyroid hormone, or incorrect parathyroid hormone levels for serum calcium values [1], and represent the third most frequent endocrinopathy after diabetes mellitus and thyroid disease [2]. PHPT affects female sex more frequently with a female to male ratio of 3–4:1 in international statistics [3] and 4.9:1 in Italy [4]. The incidence of PHPH in the general population is 27–30 cases for 100.000 peoples and often affects women older than 50 years [5]. PHPT is due to the presence of an adenoma/single-gland disease in 80–85% of cases, multiple gland disease or hyperplasia accounts for 10–15% of cases of PHPH. Atypical parathyroid adenoma (APA) and parathyroid carcinoma (PC) are both responsible for about 1.2–1.3% and 1% or less of PHPT, respectively. However, the true incidence of APA is unknown. Rarely, PHPT is due to ectopic tissue PTH production from a non-parathyroid tumor [6, 7]. APA is a lesion with suspicious clinical and histological features of malignancy, but it does not completely respect the World Health Organization (WHO) criteria for the diagnosis of PC [8]. The main purpose of this study is to identify clinical, laboratory and histological differences in patients with typical adenoma (TA) and APA and to evaluate the risk of disease recurrence in patients with APA.

## Materials and methods

We enrolled 117 patients from August 2010 to November 2018 undergoing parathyroidectomy for PHPT at the General Surgery Operative Unit (ASST Spedali Civili-University of Brescia, Italy). Histological and immunohistochemical examination showed that 107 patients (91.5%) were affected by TA (group A), while 10 patients (8.5%) were affected by APA (group B). None of the patients were affected by PC. Written and verbal information was given to the patients before enrolment, and written informed consent was obtained. The study was conducted in full accordance with the World Medical Association Declaration of Helsinki.

### Inclusion criteria

The retrospective study included patients over the age of 18 undergoing parathyroidectomy with symptomatic or asymptomatic PHPT with surgical indication according to expert opinion and guideline [9–12].

### Exclusion criteria

Patients undergoing parathyroidectomy for parathyromatosis, secondary and tertiary hyperparathyroidism, and familiar PHPT are the exclusion criteria. We also excluded patients undergoing parathyroidectomy for PHPT with an incomplete family history.

### Preoperative and postoperative management and surgical technique

All patients were investigated preoperatively by single proton emission computed tomography (SPECT/CT) to allow better capacity for discrimination of the parathyroid glands and to facilitate the surgical approach [13]. A neck ultrasound color-Doppler imaging (neck US-CD) was performed on all patients with high-resolution B-mode ultrasound machines following a standardized protocol [14]. Patients underwent abdominal ultrasound color-Doppler imaging (abdominal US-CD) for the d69 + detection of nephrolithiasis or nephrocalcinosis. The surgical technique performed was minimally invasive parathyroidectomy (MIP) with adjunctive intraoperative PTH monitoring (IPM) according to Miami protocol [15, 16]. IPM was used to evaluate the adequacy of parathyroidectomy [14]. Conversion to bilateral exploration (BE) intervention was adopted when IPM was suggestive for hypersecretion from residual parathyroid tissue [17, 18]. Short-term supplementation for prophylaxis against hypocalcemia with vitamin D and calcium carbonate was administered to all patients after MIP. A long-term follow-up and iPTH assay at 2 weeks and at 6, 12, 24, 36, 48, and 60 months was done during the post-operative course. Persistent PHPT was considered as a failure to obtain normal calcium value within 6 months after MIP. Recurrent PHPT was established as a finding of hypercalcemia 6 months after MIP. Hypoparathyroidism following surgery was classified as temporary or permanent when a drug administration is required for longer than 12 months after surgery [9, 19].

### Anthropometric assessment and laboratory measurements

Serum calcium (mg/dL), 24 h urinary calcium (mg/24 h), and creatinine (mg/dL) were measured using standard automated techniques. Modification of diet in renal disease (MDRD) (mL/min/1.73 m^2^) formula was used to calculate the glomerular filtration rate (eGFR) [20]. Parathyroid hormone (iPTH) (pg/mL) was tested with a two-site assay that measured “intact” hormone and 25-hydroxyvitamin D (25-OH-vitamin D) (ng/mL) was tested using the radio-immunoassay technique.

### Anatomic pathology analysis

Tissue samples were macroscopically (described, measured, weighed), histologically, and immunohistochemically analyzed. APA was defined as a neoplasm that exhibits some of the features of PC but lacks unequivocal invasive growth, vascular/perineural invasion, soft tissue, or surrounding structures (including thyroid, recurrent laryngeal nerve, trachea, esophagus) invasion or documented metastatic disease, according to WHO criteria for malignancy.

Macroscopic features include clinical/intraoperative adherence and presence of thick capsule, pseudocapsular invasion, bands of fibrosis (with or without associated hemosiderin deposition), pronounced trabecular growth, nuclear atypia, prominent nucleoli, and mitotic activity (> 1/10 high-power fields (HPFs). In order to evaluate malignancy, we assessed proliferation index (MIB-1/Ki-67 greater than 1% and equal or less than 4%) and vascular invasion (CD31 and CD34). We determined the immunohistochemical profile of APA versus TA, by testing PTH expression, galectin-3. APA was positive for a protein gene product (PGP) 9.5 with diffuse strong positive staining in more than 50% of tumor cells. APA was also considered positive for galectin-3 when more than 30% of tumor cells are positive.

### Statistical analyses

Data analysis and management were performed using IBM® SPSS® Statistics 20 for Windows® software. A probability value of *p* <  0.05 was considered to be statistically significant. The normality of variables was tested using the Shapiro-Wilk method for normal distributions. All continuous variables were expressed as mean ± standard deviation, and categorical variables were expressed as numbers (percentage). Fisher’s exact test was used for the comparison of categorical data. Mann–Whitney *U* test was performed to determine differences between groups.

## Results

**Fig. 1:**
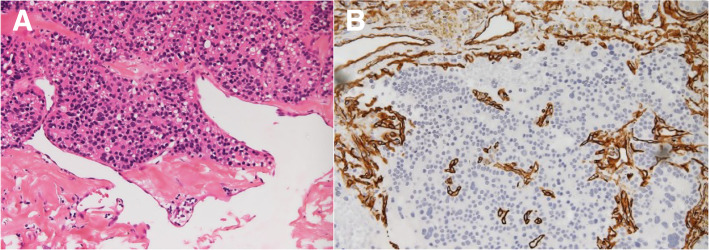
**a** Pseudocapsular invasion: cell proliferation is closer with capsular and vascular structures in the atypical parathyroid adenoma but there is no sure invasion (hematoxylin and eosin (H&E) staining, × 40 original magnification). **b** Pseudocapsular invasion: immunohistochemical staining for CD31/CD34 underlining the vascular plot allows to verify if there is an invasion of tumor cells and therefore improve diagnostic quality (× 40 original magnification)

**Fig. 2:**
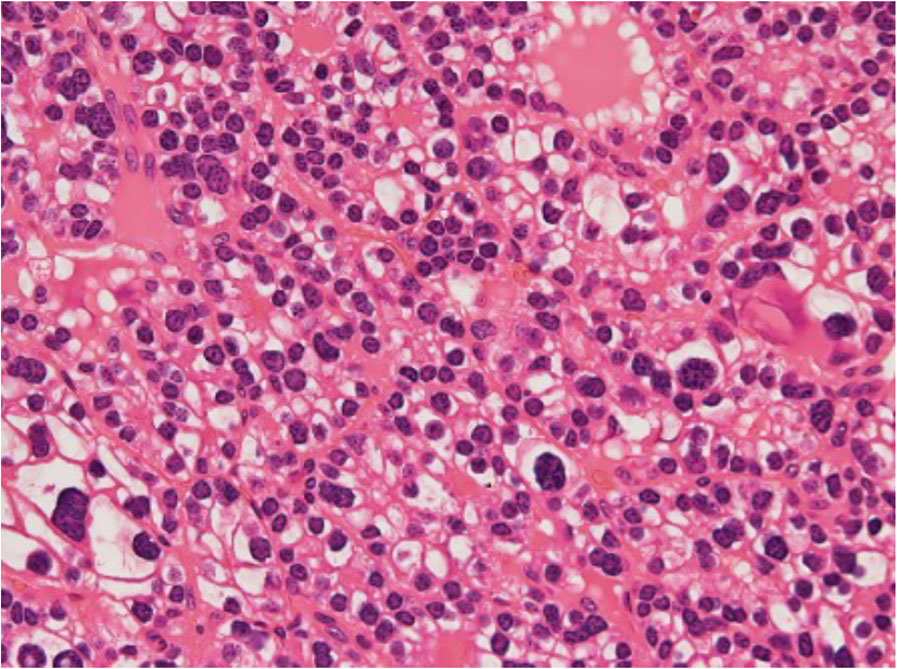
Nuclear pleomorphism: characteristic dosimetry and hyperchromasia of tumor cells nuclei in high-power view section of atypical parathyroid adenoma (H&E staining, × 100 original magnification)

**Fig. 3:**
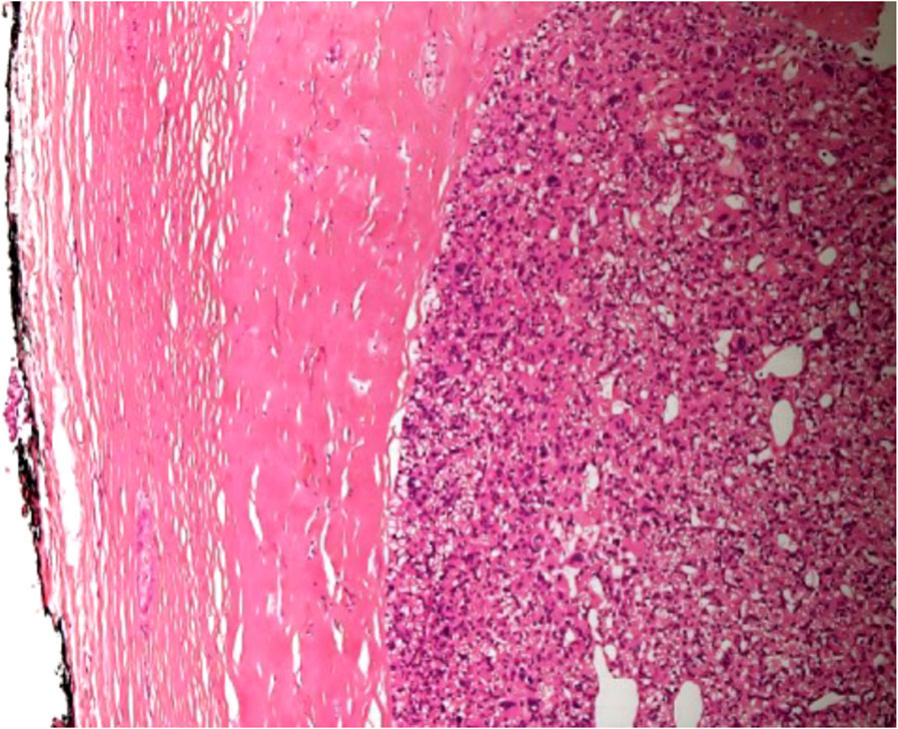
Thick capsule: a characteristic of the atypical parathyroid adenoma is also present in parathyroid carcinoma; therefore, it requires a strict differential diagnosis (H&E, × 40 original magnification)

**Fig. 4:**
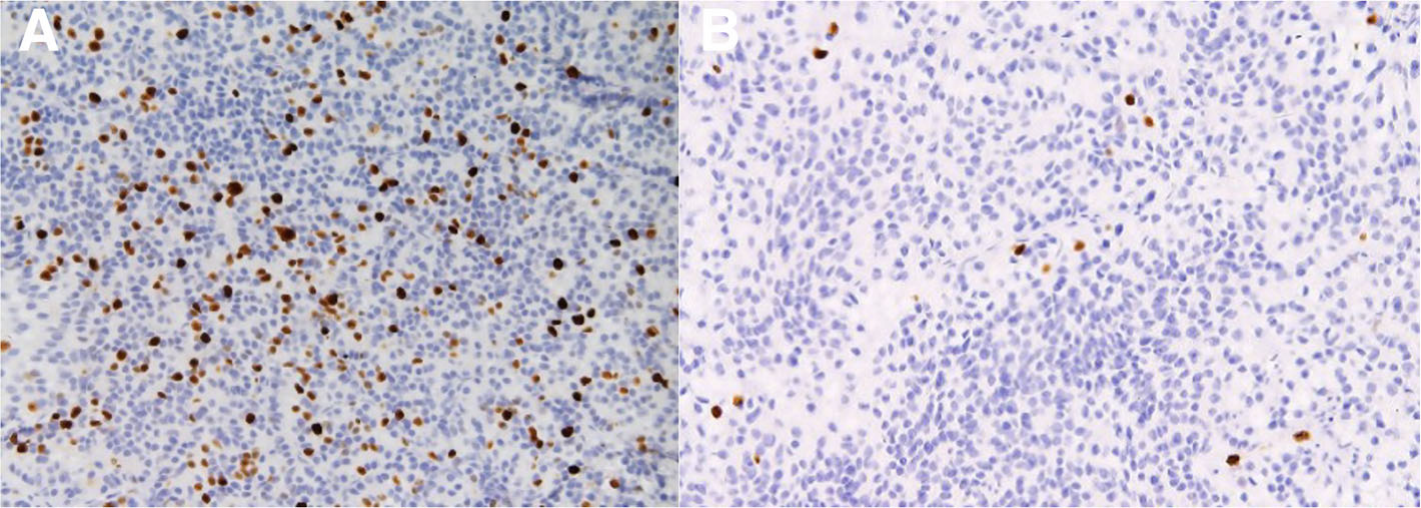
**a** Ki-67+ > 4%: immunohistochemical staining for Ki-67 underlines the high mitotic rates of APA (MIB/Ki-67, × 40 original magnification). **b** Ki-67+ < 4%: immunohistochemical staining for Ki-67 underlines the low mitotic rates of TA (MIB/Ki-67, × 40 original magnification)

**Fig. 5:**
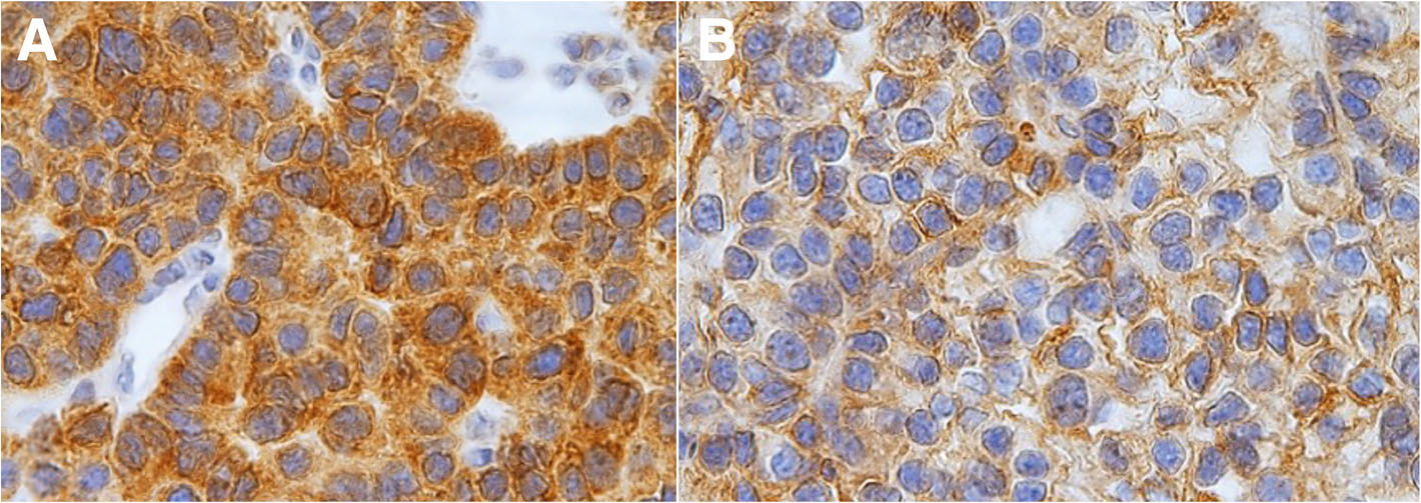
**a** Galectin-3+: immunohistochemistry for Galectin-3 shows widespread and marked cytoplasmic and nuclear staining of APA (× 400 original magnification). **b** Galectin-3 +: immunohistochemistry for galectin-3 shows weakly cytoplasmic and nuclear staining of TA (× 400 original magnification)

Baseline characteristics of patients and biochemical analyses before and after MIP are shown in Table 1. A total of 117 patients (35 males) with a mean age of 60.4 ± 13 years were enrolled. The mean follow-up time was 60 ± 1 month (with a median follow-up time of 60 months and a follow-up time range of 60–61 months). Symptomatic hypercalcemia was present in 19 patients (16.2%) at the diagnosis. Preoperative iPTH was 459.2 ± 602.1 pg/ml before MIP. Transient hypoparathyroidism was found in 7 patients after MIP. None of the patients showed permanent hypoparathyroidism or persistent and/or recurrence hyperparathyroidism during follow-up. Anatomic pathology findings in the overall population after MIP are shown in Table 2. Histological and immunohistochemical examination showed that 107 patients (91.5%) were affected by TA (group A), while 10 patients (8.5%) were affected by APA (group B). Seven patients (6.0%) had double TA. None of the patients were affected by PC (0.0%). Comparison between group A and group B are shown in Table 3. No significant differences were found between the two groups regarding sex, age, and body mass index (BMI) (*p* = 0.105, *p* = 0.661, *p* = 0.257). The percentage of patients with symptomatic hypercalcemia and fragility fracture at the diagnoses was significantly higher in patients with APA (*p* = 0.001; *p* = 0.019). Preoperative serum calcium and iPTH values were significantly lower in group A with respect to group B (11.7 ± 2.1 pg/ml vs 13.6 ± 1.5 pg/ml, *p* = 0.001; 390.1 ± 473.6 pg/ml vs 1198.7 ± 1168.7 pg/ml, *p* = 0.002). Twenty-four patients (22.4%) had a T score ≤ 2.5 compatible with osteoporosis in group A, while 3 patients (30%) presented osteoporosis in group B (*p* = 0.695). Thirty-six patients (30.8%) suffering of nephrolithiasis in group A and 2 patients (20%) in group B (*p* = 0.722). The percentage of patients with peptic ulcers, pancreatitis, cardiovascular disease, hypertension, neuropsychiatric/neurocognitive symptoms, neuromuscular symptoms, CrCl < 60 ml/min other comorbidities were not statistically significant differences between the 2 groups. Intraoperative iPTH value was higher in group B with respect to the group A, but the difference was not statistically significant (*p* = 0.108). There were not statistically significant differences in serum calcium values of I, II, and III postoperative days (*p* = 0.488; *p* = 0.111; *p* = 0.747). Also, there were no statistically significant differences between postoperative iPTH and 6 months of iPTH values during follow-up (*p* = 0.099; *p* = 0.696). Anatomic pathology findings comparison between TA and APA are shown in Table 4. APA presented with greater size (*p* = 0.001) and weight (*p* <  0.001). The two groups presented significant statistical differences in histological and immunohistochemical parameters as pseudocapsular invasion (*p* <  0.001, Fig. 1a, b), bands of fibrosis (*p* <  0.001), pronounced trabecular growth (*p* < 0.001), mitotic rates of > 1/10 HPFs (*p* < 0.001), nuclear pleomorphism (*p* = 0.036, Fig. 2), thick capsule (*p* < 0.001, Fig. 3), Ki-67+ > 4% (*p* < 0.001, Fig. 4a, b), galectin-3+ (*p* = 0.002, Fig. 5a, b), PGP 9.5 + (*p* = 0.038). We did not find any disease recurrence in the 10 patients with APA during the follow-up period.

**Table 1:** Baseline characteristics of patients and biochemical analyses before and after MIP

	Total population
	*n* = 117
Male, *n* (%)	35 (29.9)
Age (year)	60 ± 13
BMI (kg/m^2^)	25.5 ± 4.0
Symptomatic hypercalcemia	19 (16.2)
Nephrolithiasis	36 (30.8)
Peptic ulcers	3 (2.6)
Pancreatitis	4 (3.4)
Cardiovascular disease	18 (15.4)
Hypertension	55 (47.0)
Osteoporosis	27 (23.1)
Fragility fracture	3 (2.6)
Neuropsychiatric/neurocognitive symptoms	7 (6.0)
Neuromuscular symptoms	6 (5.1)
CrCl < 60 ml/min	11 (9.4)
Others symptoms	16 (13.7)
Serum creatinine (mg/dL)	1.2 ± 0.7
eGFR (mL/min)	89.2 ± 38.1
Preoperative serum calcium (mg/dL)	11.8 ± 2.1
25-OH vitamin D (ng/mL)	53.5 ± 15.6
24-h urinary calcium	279.1 ± 112.1
Preoperatve iPTH (pg/ml)	459.2 ± 602.1
Intraoperative iPTH (pg/ml)	59.7 ± 71.1
Postoperative iPTH (pg/ml)	25.3 ± 17.3
6 months iPTH (pg/ml)	23.0 ± 9.8
I postoperative day serum calcium (mg/dL)	9.1 ± 0.6
II postoperative day serum calcium (mg/dL)	9.0 ± 0.6
III postoperative day serum calcium (mg/dL)	8.9 ± 0.5
Follow-up time (months)	60 ± 1
Temporary hypoparathyroidism	7 (6.0)
Permanent hypoparathyroidism	0 (0.0)
Persistent hyperparathyroidism	0 (0.0)
Recurrence hyperparathyroidism	0 (0.0)
Disease relapse	0 (0.0)

Data are shown as mean ± standard deviation or number (%)

*Abbreviations*: *MIP* minimally invasive parathyroidectomy, *BMI* body mass index, *CrCl* creatinine clearance, *eGFR* estimated glomerular filtration rate, *25-OH vitamin D* 25-hydroxyvitamin D3, *iPTH* intact-parathyroid hormone, *PTX 6 months iPTH* intact-parathyroid hormone measurement 6 months later the surgery intervention

**Table 2:** Anatomical pathology findings in the overall population

	Total population
	*n* = 117
Single gland disease	110 (94.0)
Typical adenoma	100 (85.5)
Atypical parathyroid adenoma	10 (8.5)
MGD	7 (6.0)
Double typical adenoma	7 (6.0)
Parathyroid hyperplasia	0 (0.0)
Parathyroid carcinoma	0 (0.0)
Size of parathyroid gland (mm)^a^	24 ± 14
Weight of parathyroid gland (mg)	33 ± 42
Clinical/intraoperative adherence	0 (0.0)
Pseudocapsular Invasion	4 (3.4)
Bands of fibrosis	9 (7.7)
Pronounced trabecular growth	13 (11.1)
Mitotic rates of > 1/10 HPFS	8 (6.8)
Tumor necrosis^b^	1 (0.9)
Nuclear pleomorphism	4 (3.4)
Cystic degeneration	10 (8.5)
Thick capsule	4 (3.4)
Hemosiderin deposition	5 (4.3)
Hematic extravasion	7 (6.0)
Ki-67+ < 1%	11 (9.4)
Ki-67+ > 4%	4 (3.4)
Galactin-3 +	29 (24.8)
PGP 9.5 +	17 (14.3)
Criteria for malignancy^c^	0 (0.0)

Data are show as mean ± standard deviation or number (%)

*Abbreviations*: *MGD* multiglandular disease, *HPFS* high-power fields

^a^Maximum diameter

^b^Not associated with tumorous infarction or fine-needle aspiration

^c^Invasion of capsule, vascular, perineural, soft tissue, or surrounding structures (including thyroid, recurrent laryngeal nerve, trachea, esophagus) or documented metastatic disease

**Table 3:** Comparison between patients diagnosed with TA (group A) and patients diagnosed with APA (group B)

	Group A typical adenoma	Group B atypical adenoma	***P-value***
	*n* = 107	*n* = 10	
Male n (%)	30 (28.0)	5 (50.0)	*0.105*
Age (year)	60 ± 13	62 ± 13	*0.661*
BMI (kg/m2)	25.4 ± 4.0	26.4 ± 3.6	*0.258*
Symptomatic hypercalcemia	13 (12.1)	6 (60.0)	*0.001*
Nephrolithiasis	34 (31.8)	2 (20.0)	*0.722*
Peptic ulcers	2 (1.9)	1 (10.0)	*0.237*
Pancreatitis	3 (2.8)	2 1.9)	*0.304*
Cardiovascular disease	16 (15.0)	2 (20.0)	*0.651*
Hypertension	53 (49.5)	2 (20.0)	*0.101*
Osteoporosis	24 (22.4)	3 (30.0)	*0.695*
Fragility fracture	1 (0.9)	2 (20.0)	*0.019*
Neuropsychiatric/neurocognitive symptoms	6 (5.6)	1 (10.0)	*0.474*
Neuromuscular symptoms	5 (4.7)	1 10.0)	*0.422*
CrCl < 60 ml/min	10 (9.3)	1 (10.0)	*1.000*
Others symptoms	14 (13.1)	2 (20.0)	*0.625*
Serum creatinine (mg/dL)	1.2 ± 0.7	1.1 ± 0.7	*0.750*
eGFR (mL/min)	89.2 ± 38.7	89.9 ± 33.3	*0.884*
Preoperative serum calcium (mg/dL)	11.7 ± 2.1	13.6 ± 1.5	*0.001*
25-OH vitamin D (ng/mL)	53.2 ± 15.8	56.8 ± 13.4	*0.372*
24-h urinary calcium	269.9 ± 102.8	376.8 ± 160.7	*0.036*
Preoperatve iPTH (pg/ml)	390.1 ± 473.6	1198.7 ± 1168.7	*0.002*
Intraoperative iPTH (pg/ml)	56.6 ± 68.9	93.1 ± 88.9	*0.108*
Postoperative iPTH (pg/ml)	24.8 ± 16.8	31.4 ± 22.2	*0.099*
6 mounths iPTH (pg/ml)	23.0 ± 10.0	22.3 ± 6.4	*0.696*
I Postoperative day serum calcium (mg/dL)	9.1 ± 0.6	9.2 ± 0.6	*0.488*
II Postoperative day serum calcium (mg/dL)	9.1 ± 0.6	8.9 ± 0.6	*0.111*
III Postoperative day serum calcium (mg/dL)	8.9 ± 0.4	8.8 ± 0.6	*0.747*
Follow-up time (mounths)	60 ± 2	61 ± 4	*0.551*
Temporary hypoparathyroidism	6 (5.6)	1 (10.0)	*0.474*
Permanent hypoparathyroidism	0 (0.0)	0 (0.0)	*–*
Persistent hyperparathyroidism	0 (0.0)	0 (0.0)	*–*

Data are shown as mean ± standard deviation or number (%)

*Abbreviations*: *TA* typical adenoma, *APA* atypical parathyroid adenoma, *BMI* body mass index, *CrCl* creatinine clearance, *eGFR* estimated glomerular filtration rate, *25-OH vitamin D* 25-hydroxyvitamin D3, *iPTH* intact-parathyroid hormone, *PTX 6 months iPTH* iPTH measurement 6 months later the surgery intervention

**Table 4:** Anatomical pathology findings comparison between TA (group A) and APA (group B)

	Group A typical adenoma	Group B atypical adenoma	***P value***
	*n* = 107	*n* = 10	
Single gland disease	100 (93.5)	10 (100.0)	*1.000*
Double typical adenoma	7 (6.5)	0(0.0)	*1.000*
Size of parathyroid gland (mm) ^a^	23 ± 13	25 ± 30	*0.001*
Weight of parathyroid gland (mg)	40 ± 16	120 ± 64	*< 0.001*
Clinical/intraoperative adherence	0 (0.0)	0 (0.0)	*–*
Pseudocapsular invasion	0 (0.0)	4 (40.0)	*< 0.001*
Bands of fibrosis	4 (3.7)	5 (50.0)	*< 0.001*
Pronounced trabecular growth	7 (6.5)	6 (60.0)	*< 0.001*
Mitotic rates of > 1/10 HPFS	2 (1.9)	6 (60.0)	*< 0.001*
Tumor necrosis^b^	0 (0.0)	1 (10.0)	*0.085*
Nuclear pleomorphism	2 (1.9)	2 (20.0)	*0.036*
Cystic degeneration	8 (7.5)	2 (20.0)	*0.204*
Thick capsule	0 (0.0)	4 (40.0)	*< 0.001*
Hemosiderin deposition	3 (2.8)	2 (20.0)	*0.058*
Hematic extravasion	5 (4.7)	2 (20.0)	*0.110*
Ki-67+ < 1%	11 (10.3)	0 (0.0)	*0.598*
Ki-67+ > 4%	0 (0.0)	4 (40.0)	*< 0.001*
Galactin-3 +	22 (20.6)	7 (70.0)	*0.002*
PGP 9.5 +	13 (12.1)	4 (40.0)	*0.038*

Data are show as mean ± standard deviation or number (%)

*Abbreviations*: *HPFS* high-power fields

^a^Maximum diameter

^b^Not associated with tumorous infarction or fine-needle aspiration

## Discussion

APA is a rare cause of PHPT responsible for approximately 1.3–1.2% of cases. It represents a difficult diagnosis for the Surgeon and the Pathologist, particularly in the differential diagnosis versus PC. In our study, the age of presentation of about 60 years was similar between APA and TA, a point highlighted in other studies [6, 21, 22]. APA presented a 1:1 male/female ratio similar to that found in PC [1]. The effects of menopause, hormonal influence, and gene deletions that predispose female sex to TA development do not seem to be relevant in the pathogenesis of APA and PC [23]. The serum calcium level was higher in patients with APA than in patients with TA in accordance with literature data [6]. Furthermore, we found a higher incidence of symptomatic hypercalcemia in APA similar to PC, as reported by Fernandez-Ranvier et al. [24]. The high preoperative levels of serum calcium and iPTH characteristic of PC are a confounding element that complicates differential diagnosis. We found no significant differences in intraoperative and postoperative iPTH values and in serum calcium in I, II, and III postoperative days as reported by Bilezikian et al. [11]. In our study, the incidence of nephrolithiasis and neurocognitive symptoms was similar to that reported in the literature [4, 25]. Patients with hyperparathyroidism often suffering from asthenia, depression, difficulty concentrating, anxiety, and neurocognitive decay. The pathogenesis of neurocognitive disorders in PHPT is unclear and perhaps may be associated with the key role of calcium in neurotransmission signals [4]. We also reported a lower incidence of osteoporosis and pathological fractures than reported in 2018 by the Italian Society of Endocrinology [25]. None of our patients had a palpable neck mass, as also referred by McCoy and Fernandez-Ranvier [6, 24]. It is probably due to an early clinical diagnosis and a wider use of calcium and iPTH screening in the general population. Single-photon emission computed tomography (SPECT/CT) and Neck US-CD are the most frequently used instrumental examinations for preoperative disease localization as suggested by the guidelines [4, 9, 13, 18, 26]. Patel et al. [27] also reported that the combined use of SPECT-CT and Neck US/CD in the same patient can improve sensitivity for disease localization up to 95%. In our study, APA was greater and heavier than TA as also reported by Agarwal [28] and O’Neal [22]. Furthermore, we found several histological and immunohistochemical differences in patients with APA compared to patients with TA (Table 4). According to Fernandez-Ravier et al. [24], we found some histopathological differences in APA than TA as pronounced trabecular growth (*p* < 0.001), mitotic rates of > 1/10 HPFs (*p* < 0.001), nuclear pleomorphism (*p* = 0.036, Fig. 2), thick capsule (*p* < 0.001, Fig. 3). Agarwal et al. [28] reported a lower positivity of galectin-3 (47.4%) in patients with APA than in our study (70%). Perhaps this difference is probably due to the limited number of samples of both studies. In fact, Cetani et al. [29] in a recent systematic review of the literature finds a range of overexpression between 32 and 100%. Some studies underline the role of loss of nuclear parafibromin immunoreactivity in PC and APA. Nuclear parafibromin loss is rarely in PA. A significant heterogeneity of loss of nuclear staining was reported in APA (from 0 to 50%). Parafibromin marker appears to be useful in the differential diagnosis between PC and APA, but its role in APA remains unclear. Other markers such as galectin-3 and MIB/Ki 67 seem to be similar in the differential diagnosis between APA and PA [29–31]. Parafibromin marker was not used in our study. Currently, the most reliable criteria for the diagnosis of APA are associated with a careful histological and immunohistochemical examination. Differential diagnosis between PA and APA requires a number of sections suitable to exclude the presence of criteria of malignancy. Therefore, morphological and immunohistochemical methods supporting the diagnosis seem necessary and essential. Patients with APA require careful follow-up. In the mean follow-up of 60 months, we did not find any relapse in the 10 patients with APA (Table 1), also Chiu et al. reported a percentage of relapses of 0% [15], while Kruijff et al. reported 3.70% [31]. We believe this difference depends on the experience of the pathologist [32, 33] and anatomical pathology methods, although the limited follow-up could have affected the result.

## Conclusions

APA is a tumor that has characteristics both of TA and PC. The diagnosis is reached by excluding the presence of malignancy criteria. APA compared to the TA showed significant clinical, hematochemical, histological, and immunohistochemical differences. Therefore, considering the uncertain future that accompanies this tumor, APA should be treated as a “Tumor of Uncertain Malignant Potential”, and we suggest for these patients a careful and strict follow-up.

### Limitations of the study

The limitation of our study is mainly represented by the relatively small size of the APA group. Additional studies are necessary with a larger sample and longer follow-up to confirm our results.

## Data Availability

Data used are available from the authors upon request.
